# Healthcare Professionals’ Views of Factors Influencing Diabetes Self-Management and the Utility of a mHealth Application and Its Features to Support Self-Care

**DOI:** 10.3389/fendo.2022.793473

**Published:** 2022-02-24

**Authors:** Sungwon Yoon, Jun Hao Ng, Yu Heng Kwan, Lian Leng Low

**Affiliations:** ^1^ Health Services and Systems Research, Duke-NUS Medical School, Singapore, Singapore; ^2^ Centre for Population Health Research and Implementation, SingHealth Regional Health System, Singapore, Singapore; ^3^ Duke-NUS Medical School, Singapore, Singapore; ^4^ Department of Pharmacy, National University of Singapore, Singapore, Singapore; ^5^ Internal Medicine Residency Programme, SingHealth Residency, Singapore, Singapore; ^6^ Department of Family Medicine and Continuing Care, Singapore General Hospital, Singapore, Singapore; ^7^ Post-Acute and Continuing Care, Outram Community Hospital, Singapore, Singapore; ^8^ SingHealth Duke-NUS Family Medicine Academic Clinical Program, Singapore, Singapore

**Keywords:** diabetes, self-management, healthcare professional, mobile health application, behavior change

## Abstract

**Introduction:**

The perspectives of healthcare professionals (HCPs) are pivotal to co-development of self-management strategies for patients with diabetes. However, literature has been largely limited to perspectives of patients within the context of a Western healthcare setting. This study aims to explore factors influencing diabetes self-management in adult patients with diabetes from the perspectives of HCPs and their views of the value of mHealth application for diabetes self-management.

**Materials and Methods:**

We conducted focus group discussions (FGD) with purposively selected HCPs in Singapore. All FGDs were audio-recorded and transcribed verbatim. Thematic analysis was conducted using NVivo 12.

**Results:**

A total of 56 HCPs participated in the study. Barriers to self-management included limited patient commitment to lifestyle changes, suboptimal adherence to medication and treatment, patient resistance to insulin initiation and insufficient rapport between patients and HCPs. Patients’ perceived susceptibility to complications, social support from family and community, multidisciplinary team care and patient’s understanding of the benefits of self-care were viewed as facilitating self-management. HCPs saw mHealth apps as a vital opportunity to engage patients in the self-management of conditions and empower them to foster behavior changes. Yet, there were concerns regarding patient’s limited digital literacy, lack of integration into routine electronic system and reluctance.

**Discussion:**

We identified a set of factors influencing self-management in adult patients with diabetes and useful app features that can empower patients to manage their conditions. Findings will inform the development of a mHealth application, and its features designed to improve self-care.

## Introduction

Diabetes mellitus is a chronic metabolic disease associated with serious complications and high healthcare cost, affecting 1 in 5 adults 65 years and above worldwide (1). Older patients with diabetes are at risk of developing vascular complications, due to a longer disease duration and decline of physiological reserve (2). These complications include retinopathy, nephropathy, neuropathy and heart diseases (3) which can negatively impact the quality of life and deepen the cost burden of disease. In Singapore where this study was conducted, the prevalence of diabetes (14.2%) had surpassed the global average (9.3%) in 2019 (1), and is projected to reach 25% in 2050 (4). Type 2 diabetes (T2DM) accounts for 99% of all diagnosed cases of diabetes (5). The lifetime medical expenditure of patients with diabetes was shown to be 5.6 times higher than that of patients with non-diabetes (6). Nationally, healthcare spending for diabetes is estimated to increase from US$787 million in 2010 to US$1,867 million in 2050 (7). The increased socioeconomic burden associated with diabetes underscores the importance of early intervention to prevent and delay the complications resulting from the disease.

Diabetes self-management is defined as an active participation of health-seeking behaviors and activities by patients, to attain good glycemic control and reduce complications (8, 9). The strategies for self-management include lifestyle modification (healthy diet, physical activity, weight loss) (10, 11), psychosocial support (12), education on self-management (13, 14) and use of mobile technology (15–17). Lifestyle modification is the first-line treatment for diabetes mellitus and has been shown to be more effective than pharmacotherapy (10, 11), However, lifestyle modification requires significant commitments and motivation on the part of patients to sustain behavior changes (18). A novel model of care that takes advantage of mobile health (mHealth) technology has been found to be effective in increasing patients’ interest and motivation for lifestyle modification (19). A growing body of literature also suggests that setting personalized goals, supporting self-monitoring and providing feedback on alterations in diet and physical activities through mHealth apps enhanced the ability of patient with diabetes for self-management (20–22).

Perspectives of healthcare professionals (HCPs) are pivotal to co-development of self-management strategies for patients with diabetes as HCPs can act as an enabler to empower patients through shared understanding and partnership. Existing literature reported common barriers faced by HCPs which include patient-related factors (motivation, health literacy, time constraints, finances, comorbidity, cultural differences) and HCP-related factors (heavy workload, poor patient-provider relationship) (23–27). In addition to barriers to self-management in adult patients with diabetes, recent systematic reviews highlighted that coping skills, relationship with family and peers and diabetes education may enable or hamper effective self-management in families with children and adolescents with diabetes (28, 29). Although existing studies have offered a significant insight into the factors influencing diabetes self-management, they have been largely limited to the perspectives of patients within the context of a Western healthcare setting. In addition, there is a paucity of research on perceptions of HCPs regarding potential values and utility of a mHealth application and its features in fostering self-management and improving quality care for patients with diabetes.

This study aims to explore the factors influencing self-management in adult patients with diabetes from the perspectives of HCPs in a multi-ethnic Asian healthcare setting. We also sought to understand the HCP’s perceptions of and attitudes to a mobile health (mHealth) application and its features for self-management. Findings from this study will inform the design of an optimal mHealth intervention that can foster self-management and address the needs of HCPs in caring of adult patients with diabetes.

## Materials and Methods

### Study Design and Data Collection

This study used a qualitative research method involving focus group discussions (FGDs). FGD is a qualitative research method for eliciting data from population subgroups in order to develop an understanding of perspectives on particular topics (30, 31). We conducted FGDs with HCPs to elicit their perspectives of self-management in patients with diabetes and its integration with mHealth, between May 2020 and February 2021. FGD was selected as it allows for an observation of how ideas and issues emerge and are prompted by the variety of different participants’ contributions on a topic of our interest (32). HCPs from acute care or community hospitals and primary care centers with at least one-year experience in direct care for adult patients with diabetes were invited to participate *via* email. A purposive sampling technique based on age, gender, profession, duration of practice and education was used to obtain a diverse range of opinions and experiences.

All FGDs were conducted in English by a facilitator trained in qualitative research, through online video conferencing. Each FGD comprised 2 to 5 HCPs and lasted approximately 65-100 minutes. A discussion guide was developed based on existing literature (33–36) and the study team’s expertise and pilot tested. The guide incorporated open-ended questions on different aspects of diabetes management. Each FGD was divided into two parts. First, HCPs were asked to describe their experience in managing adult patients with diabetes and elaborate on the factors they considered important for patients to self-manage their conditions. Next, HCPs were shown a demonstration of a prototype mHealth platform for self-management and asked about the features they found useful. HCPs were also prompted to indicate any missing features that were essential to self-management in patients with diabetes but was not discussed. The prototype mHealth platform, guided by behavioral change wheel (37) and nudge theory (38), consists of 5 key features: daily logs and report for diet, physical activity, blood glucose and medication; personalized nudges and reminders generated based on user’s inputs; gamification; goal setting; and educational resources. In addition, we presented several visual materials collated from existing mHealth app features. As the development of our mHealth application would be intended for adult patients with T2DM aged 40 years and above, the discussion was primarily confined to T2DM and adult patient population. The FGDs were moderated by a researcher trained in qualitative research. This study was approved by the SingHealth Centralized Institutional Review Board (Ref No: 2019/2468).

### Data Analysis

All FGDs were audio-recorded and transcribed verbatim. Thematic analysis was conducted using NVivo 12 software based on grounded theory (39). Grounded theory was chosen as it offered conceptualization of data and generation of conceptually abstract categories grounded in the data. Each FGD transcript was read line-by-line and coded independently by two coders (SY, JN). The codes were simultaneously checked by study team members to increase validity of emerging themes. Consensus was achieved through discussions and reviews of codes and categories. A list of emerging themes was compared to those generated through the subsequent transcripts. All codes were reviewed together by the study team to ensure that common themes reflected a shared understanding among participants of the phenomena under investigation. Data collection and analysis were carried out in an iterative manner until thematic saturation was reached. To improve transparency and rigor, we anchored our methodology according to the Consolidated Criteria for Reporting Qualitative Research (COREQ) checklist (40).

## Results

### Characteristics of Participants

A total of 56 HCPs (38 clinicians, 10 nurses, 4 dieticians, 4 pharmacists) participated in 18 FGDs. The age range (median) of HCPs was 27 to 55 years (34 years old). Around 80% of HCPs were Chinese and 58% of them were female. A majority of HCPs (46.4%) were from public primary care centers where a greater proportion of adult patients with diabetes in Singapore are being treated while 34% of HCPs were from diabetes centers in three tertiary hospitals which provide multidisciplinary services for diabetes management and education. The remaining HCPs (19.6%) were from dietetics services of community hospitals. Data saturation was reached after seven FGDs for clinicians and four FGDs for allied health practitioners. The demographic profile of HCPs is summarized in **Table 1**.

**Table 1:** Participant characteristics (n = 56).

	n	(%)
**Age (year)**		
Mean (range)	34	(27-55)
**Gender**		
Male	23	(41.0%)
Female	33	(58.9%)
**Ethnicity**		
Chinese	45	(80.3%)
Malay	5	(8.92%)
Indian	5	(8.92%)
Sinhalese	1	(1.78%)
**Education level**		
University degree	25	(44.6%)
Masters	19	(33.9%)
Postgraduate diploma or degree	12	(21.4%)
**Profession**		
Clinician	38	(67.8%)
Nurse	10	(17.8%)
Dietician	4	(7.14%)
Pharmacist	4	(7.14%)
**Duration of clinical experience**		
1 to 5 years	18	(32.1%)
6 to 10 years	22	(39.2%)
11 to 15 years	8	(14.2%)
16 to 20 years	7	(12.5%)
21 to 25 years	1	(1.78%)
**Type of healthcare institution**		
Public primary care	26	(46.4%)
Diabetes center in tertiary hospital	19	(34.0%)
Community hospital	11	(19.6%)

Findings were organized according to three overarching themes: barriers to self-management; facilitators of self-management; and perceptions of a mHealth application and its features.

### Theme 1: Barriers to Self-Management

#### Limited Patient Commitment to Lifestyle Changes

HCPs believed that self-management is “beyond their control” and adult patients must be responsible for their own health. Most HCPs stated that patients did not see the necessity of lifestyle changes often because of the asymptomatic nature of the disease.

*“Because many patients find that they do not have symptoms … they are literally asymptomatic so ‘well, I do have diabetes, but I do not see any effect on me right now, so why do I need to bother so much?” (Male, Clinician, HCW 10)*

HCPs saw that patients were more motivated to change behaviors if they could avoid taking medications, due to strong aversion to reliance on medication and its side effects. Other HCPs mentioned that comorbidities impeded self-management as patients had conflicting goals of care where diabetes was “not at the forefront of the patient’s concern”. HCPs felt that patients were generally not receptive to dietary changes due to personal preferences and cultural practices that made healthy eating difficult.

*“You know I had many patients who tell me stuff like they cannot live without rice, they don’t like the taste of coffee without sugar. That kind of personal sacrifices that they may not be ready to make yet.” (Male, Clinician, HCW 09)*

The situation seemed further exacerbated when access to diabetes-friendly food was denied by high cost and limited diabetes-friendly food options. Additionally, HCPs reported their encounter with patients who had no time to engage in physical activity or attend appointments due to work commitments and family priorities.

#### Suboptimal Adherence to Medication and Treatment

Non-adherence to medication was one of the main barriers identified across the FGDs hindering self-management. Non-adherence to instructions for use in terms of dose and frequency often coincided with issues with remembering capability and skills such as difficulties establishing medication routine or busy schedule. For example, HCPs reported that shift work made it difficult for some patients to adhere to medication regimen due to irregular mealtime.

*“For those who do shift work or work long hours, it’s also challenging for them to have a fixed mealtime, and as a result, mealtime cannot be fixed, and the medication also can be difficult to adjust accordingly.” (Male, Clinician, HCW 01)*

Accounts from HCPs also reflected that negative physical side effects of medications affected adherence. For example, it was reported that patients who had hypoglycemic symptoms tended to avoid taking medications or taper doses. Financial issues related to access to blood glucose monitoring apparatus was highlighted, which could hinder patients’ glycemic control since readings were required by HCPs to titrate medications.

*Patients need to buy the glucometer, lancet, test stripes and all, they have to buy them from a pharmacy out of their own pocket. It is not covered by the MediSave [national medical saving scheme] and that actually adds to the cost of treatment which is not being subsidized in anyway.” (Female, Clinician, HCW 19)*

#### Patient Resistance to Insulin Initiation

Many HCPs reported that patients declined insulin treatment, due to its perceived association with worse disease prognosis, lifelong reliance, high cost or inconvenience of self-injection.

*“I think in our population, there is this myth that if you start insulin, that means you are really bad. The not so bad patient does not need insulin but increasing dose that amounts to cost can be a barrier.” (Male, Clinician, HCW 24)*

It was also mentioned that older patients had less confidence in self-injection due to dexterity and vision impairment. HCPs described that some patients had cultural misconceptions and often made independent decisions to seek treatment from complementary medicine or herbal remedies, hoping to revert the condition.

*“They believe in some supplement that could help with the diabetes like some cooling herbal tea rather than taking the medications from the hospital or if they require insulin injection daily. They would prefer to go with their beliefs.” (Female, Nurse, HCP 42)*

#### Insufficient Rapport Between HCPs and Patients

Limited rapport building, resulting from poor communication and interactions between patients and providers, was pointed out as a factor impeding self-management. Notably, the feelings seemed mutual; HCPs labeled certain patients as “problematic” patients who invoke a sense of frustration in them, and at the same time, they acknowledged that patients may experience difficulties in their relationship with HCPs as HCPs could be seen as impersonal or lacking genuine empathy for patients’ illness.

*“They [patient] do not listen to us simply because we do not listen to them, we do not hear them out. I think they could sense that we are not trying to connect with them on a personal level, we are just being professional with them.” (Male, Clinician, HCW 01)*

HCPs in outpatient setting reported challenges in building rapport and earning trust owing to short consultation time, often being truncated by high workload and the absence of care continuity as they did not usually see the same patients again.

*“I mean in public outpatient primary care. Sometimes it’s impossible to keep seeing the same patient. That’s also a barrier because it becomes very difficult to just see that one patient.” (Male, Clinician, HCW 10)*

Thus, HCPs recognized that patients may lose trust in HCPs or the health system when there was no consistent message across HCPs during each visit.

### Theme 2: Facilitators of Self-Management

#### Perceived Susceptibility

HCPs stated that being more cognizant of diabetes complications served as positive reinforcement to motivate patients to be more proactive in self-management. HCP described that those asymptomatic patients who had witnessed suffering of their family members or friends with diabetic complications could better relate to the gravity of their own condition.

*“Having family members with diabetes or and having seen them managing well are very strong motivators for patients to have good control. Of course on the flip side, having family members who had kidney failure or stroke and heart attack relating to the diabetes also become a motivator to prevent that or to do better.” (Male, Clinician, HCW 24)*

They recounted how they had leveraged on these life experiences to educate patients on the consequences of poorly controlled diabetes. HCPs felt that successful self-management stories could further boost patients’ desire to achieve a better control of conditions.

#### Social Support From Family and Community

Patients’ relationships with family and friends and community support (support groups, community nurses) were commonly described by HCPs as having greater positive influences on patients than HCPs.

*“We [HCP] may only encounter the patient for those twenty minutes, but the patients will spend the rest of their time with their family members. If you can get them in to either motivate, remind, accompany them for follow-ups, they may be more encouraged to manage conditions.” (Female, Clinician, HCP 03)*

HCPs acknowledged that such relationships and support could empower patients by encouraging them to monitor their own health. There was consensus that family represented a source of motivation, as patients yearned to witness the milestones of their family members or preferred to be self-reliant. HCPs also saw home nursing as a bridge for the continuum of care from an inpatient to an outpatient environment.

*“For diabetes patients with complications, it is important for community nurses to monitor and track them at home as post-discharge care. When they are being adequately care for, then they can be empowered and motivated to self-manage their disease.” (Female, Nurse, HCW 49)*

#### Multidisciplinary Team Care

Many HCPs noted that a multidisciplinary approach would be beneficial to self-management, as the team could provide an extensive range of services and education to address different needs of the patient. HCPs suggested that each team member should form a voice to counsel the patients and represent an avenue of support that the patient could rely on.

*“It always helps if I discuss it with the other members in the team, anyone who had an interaction with the patient, and it gives us a perspective which I think we may be blind to, and certainly we all are at different stages of our lives and experiences, and sometimes you may offer a perspective which you did not quite see right.” (Male, Clinician, HCW 02)*

#### Understanding the Benefits of Self-Management

HCPs expressed that improving health literacy of patients could act as a catalyst to appreciate the importance of having a good glycemic control, despite being asymptomatic, and foster self-management.

*“Once, they make the connection between quality of life and DM management. They actually will try to be more compliant to medications and DM management.” (Female, Nurse, HCP 51)*

They also noticed that when blood glucose readings improved, patients appeared to be more encouraged to do self-management. Dieticians mentioned that education for self-management could be improved if information would be tailored to patients’ profile and be provided over a few sessions for easy assimilation.

*“We will try to not squeeze all the information in one session. Patient will tend to lose focus over the time. So, we would rather just focus on the few things that they need to do, at this time.” (Male, Dietician, HCW 40)*

### Theme 3: Perceptions of mHealth Application and Its Features

#### Perceived Impact of mHealth App on Diabetes Self-Management

HCPs foresaw the role of mHealth apps that could potentially address patients’ reticence to change lifestyle behaviors through shared decision-making. They felt that the active role of monitoring and tracking of one’s own indicators available on the mHealth app could empower patients to take ownership of their health, as opposed to being a passive recipient of care.

*“They would see it as a partnership about how we can change their disease in a way that can benefit them. What is the active role that they can take in that process” (Male, Clinician, HCP 12)*

Many HCPs agreed that mHealth apps could improve patient care by sharing medical records across different service providers. However, they also cautioned that apps could not replace face-to-face interaction that would be important to developing rapport between providers and patients.

HCPs noted that some patients may not possess smartphone, lack digital literacy skills or have vision and hearing impairment. Therefore, the mHealth app may be more useful for a self-selected group of patients that remains motivated, not for those who are resistant to behavior changes. HCPs also mentioned that mobile apps may have a diminished value for patients living with multiple chronic conditions that require complex care and holistic management.

*“I feel like the utility may be limited because you still need to assess the patient as a whole. So, if the portal is only going to be on diabetes it would be hard to assess the patient holistically.” (Female, Clinician, HCW 09)*

HCPs had mixed views about anticipated workload as a result of the integration of a mHealth app into their daily practice. Some HCPs indicated that if patient’s data would be prefilled prior to consultation, the mHealth app could potentially improve workflow and HCP feedback. In contrast, others were concerned about patients’ expectation to review every field that they tracked, which may lead to an additional consultation time in a busy clinic.

#### Utility of mHealth App Features

By and large, HCPs appreciated mHealth app features that support self-management in patients with diabetes. Many felt that mHealth app features could improve quality of care coordination and advance self-management. In particular, they appreciated an in-app reminder function specific to meal timings as it can ameliorate medication non-compliance by addressing many medication-related issues such as forgetfulness and incidence of side effects.

*“It would be valuable since personalized reminder can indicate if patient is on pre-meal medication, post-meal medication.” (Female, Nurse, HCW 51)*

One new feature proposed by HCPs was a function to notify HCPs to arrange for an appointment or delivery of medication refill, when a patient’s supply is running low. HCPs suggested a feature that takes advantage of social support for increased efficiency and improved health outcomes. For example, caregivers or the next-of-kin could be notified by the mobile app to respond to any irregularities in the data logged by patients such as hypoglycemic blood glucose readings.

HCPs also suggested features that may improve patients’ health literacy: one was a self-check questionnaire that enables patients to identify gaps in their diabetes knowledge after receiving in-app health education. Another suggestion was a feature that could provide patients with feedback on unhealthy food choices, by correlating data from the food diary and blood glucose trends.

*“It will be useful to see when patient’s sugar level exceeds particular limit. On the chart, if there is an icon where you can click to see the meals that patient take every time the patient’s sugar is high, then I can advise patients on diet” (Female, Clinician, HCW 26)*

HCPs expressed that app features should lessen user burden to improve adherence to data logging, for instance, automated synchronization between mHealth app, fitness trackers and glucometers and use of photographs for logging of food intake. They appreciated features that could be customized according to the needs of the patients such as personalized reminders and goal settings.

Despite perceived usefulness of mHealth app features for self-management in patients, HCPs maintained that information on the mHealth app could be incomplete, which may result in having to refer to an institution-based clinical system. These extra tasks were regarded by some HCPs as generating duplicated work.

*“I still need to manage in context of the patient. Rather than just the diabetic aspect of the patient. no matter how much an app like this is enhanced. It still cannot substitute for my Citrix [institution] app, where I can see the other parameter of the patient.” (Male, Clinician, HCP 28)*

While HCPs acknowledged the value of a two-way real-time communication with patients, which can allow patients to ask questions and clarify any misconceptions beyond the consultation, many did not endorse such function, as it would blur the boundaries of care and increase medical liability. There was a concern that patients may have a false impression or heightened expectation that HCPs are on stand-by and ready to handle an influx of queries and cases.

*“I think that would open up a bit of an ethical kind of problem. Because you can get patients who will communicate with you all times of the day. And then we do not have boundaries … at which point are we legally obligated to provide care for them.” (Female, Clinician, HCW 26)*

## Discussion

This study elucidated the factors that could impede or facilitate self-management in adult patients with diabetes from the perspectives of HCPs and their perceptions of the potential utility and value of a mHealth app and its features for diabetes self-management.

Across all professions, HCPs stressed good glycemic control as singular importance in self-management. However, they also noted that patients’ non-adherence to a prescribed schedule for meal and medication intake was the key barrier to good glycemic control. A multinational study has shown that non-adherence to treatment was a universal issue (41). For insulin initiation, patient barriers raised by HCPs in our study included cost, long-term reliance and inconvenience. This finding is in line with previous research (18, 42, 43) that patient resistance to insulin therapy is substantial and that patients generally assess the clinical efficacy of insulin as low. Importantly, HCPs in our study recognized that alternative therapies were often attempted by patients due to fear and preconceived notions of insulin risk. Several strategies should be developed to proactively address patient’s concerns and clarify misconceptions such as early discussions about insulin as a therapeutic option, engagement of patients in a shared decision making to formulate agreed target for glycemic control (44, 45) and provision of timely support to achieve effective diabetes self-management (18, 45, 46).

Our findings suggest that good rapport is perceived by HCPs to be resulting in better patient engagement and increased compliance to diabetes self-management. This finding is in line with prior literature that a good patient-provider relationship increases reception to health education and treatment compliance (25, 47, 48). Yet, our participants also felt that a short consultation time appeared to limit trust and rapport building with patients. Such time constraints were identified in earlier studies locally (49, 50) and globally (23, 51), with an average consultation time to be less than 5 minutes across different healthcare settings (52). Literature suggests that short consultation times are associated with poorer health outcomes for patients such as polypharmacy and antibiotic overuse (53, 54) whereas longer consultation length is likely to result in reduced hospital admission (52) and better disease control in patients with diabetes (55). Given the constraints of consultation time, it is often unrealistic to expect clinicians to fully engage patients for self-management. A multidisciplinary team approach, as suggested by HCPs, has been shown to be effective in improving patient care and clinical outcomes (56–58). Therefore, increased access to multidisciplinary clinics can be considered as a viable option to improve self-management.

There was agreement among HCPs that patients’ health literacy would be crucial to improving diabetes self-management. Studies reported that when diabetes patients are not familiar with their symptoms and signs, they are less likely to change lifestyle behaviors (51, 59). Conversely, it has been suggested that patients are more likely to engage in diabetes self-management if they believe they are personally susceptible to having serious diabetes complications (60). These findings, together with the findings from this study, indicate that diabetes patients will benefit greatly from personalized health education and coaching. Health education is found to be associated with increased quality of life (61) and reduced hospital admission in patients with poorly controlled diabetes (62). In light of the limited time available for patients during consultations, a novel approach incorporating mHealth apps can provide targeted health education that is timely and personalized, and thus empowers patients to monitor their conditions. Indeed, our participants unequivocally acknowledged the value of mHealth app features on health education and provision of pertinent information. Prior research showed that educational feature would enable patients to revisit information at their own pace and was shown to significantly reduce HbA1c level (63). Hence, patient-centered mHealth apps have the potential to improve self-management in diabetes patients.

HCPs in our study were generally positive about the use of mHealth app for diabetes self-management. They saw the mHealth app as an essential opportunity to engage patients in the management of conditions and empower them to adhere to lifestyle changes. However, HCPs noted that the adoption of mHealth might be hindered by high user dependency in data logging and lack of digital literacy in some patients. HCPs also anticipated a technical barrier to interoperability due to the lack of centralized IT system in public healthcare institutions. These issues resonate with prior literature which reported challenges including limited digital literacy of patients and lack of infrastructural capacity to support mHealth interventions (8, 64). Our finding underscores the importance of understanding the level of digital literacy in end users to engage them in a meaningful way and to ensure their needs are well aligned with a mHealth self-management intervention. An efficient mHealth intervention also requires an integration of mHealth platform within the existing electronic health information system.

Despite perceived utility of mHealth apps in the promotion of self-management for patients with diabetes, HCPs remained cautious about some features such as in-app communication between providers and patients. They expressed concerns about potentially engendering extra demand on their time and medical liability, as there was little bandwidth in the current model of practice in which HCP’s task is accountable by face-to-face time spent with patients in the clinic. This finding echoes that of previous studies (65, 66) that uptake of mHealth can be undermined by HCP’s perceptions of a greater workload and legal and security concerns such as patient safety and data confidentiality (67–69). It is therefore important to adequately address these concerns prior to the implementation of a mHealth app intervention. Security measures such as internet separation of IT system, user authentication, password protection and data encryption have been found useful to protect patient privacy (68, 70, 71). Medical liability associated with a two-way communication feature can be mitigated by obtaining informed consent on the boundaries of communication, before recommending an app to patients (72).

This study has contributed to an in-depth understanding of the HCP’s perceptions of the factors that influenced self-management in patients with diabetes and the utility of a mHealth application in an Asian clinical setting. Our FGDs allowed for exploration of broader contextual influences perceived by HCPs, which may have the potential to hinder or facilitate diabetes self-management. This has significant implications for a successful mHealth application. This study however has a few limitations. Although we strived to recruit a diverse range of professions, our sample was skewed with more than 60% comprising clinicians. The imbalanced sample might have introduced a bias into the study and thus influenced the theme generation. Nevertheless, we achieved data saturation in core themes. Secondly, our participants were primarily recruited from large public healthcare institutions in Singapore. Participation from HCPs practicing in the private sector may yield insights on a potentially different diabetes patient population. Lastly, since the discussion of the focus groups was largely limited to T2DM and adult patient population, our finding may not be generalizable to patients with type 1 diabetes and younger patients. Future research is warranted to understand the perspectives of HCPs on factors influencing self-management in children and adolescents with diabetes.

## Conclusion

This study identified a set of factors impeding or enabling self-management in patients with diabetes as perceived by HCPs and their views of useful app features that can empower patients to manage their conditions. Findings from this study will inform the development of a mHealth application and its features designed to improve self-care in patients with diabetes.

## Data Availability Statement

The datasets presented in this article are not readily available because of the prevailing personal data protection act and human biomedical research act. Requests to access the datasets should be directed to the corresponding author.

## Ethics Statement

This study has been reviewed and approved by SingHealth Centralized Institutional Review Board. The participants provided their informed consent to participate in this study.

## Author Contributions

SY, YK, and LL conceived and designed the study. SY and JN conducted data collection and analysis. All authors contributed to the article and approved the submitted version.

## Funding

This work was supported by the Singapore Ministry of Health’s National Innovation Challenge Grant (Ref: MOH/NIC/CDM1/2018) and Singapore Ministry of Health’s National Medical Research Council under the SingHealth Regional Health System, Population-based, Unified, Learning System for Enhanced and Sustainable (PULSES) Health Centre Grant (NMRC/CG/C027/2017_SHS).

## Conflict of Interest

The authors declare that the research was conducted in the absence of any commercial or financial relationships that could be construed as a potential conflict of interest.

## Publisher’s Note

All claims expressed in this article are solely those of the authors and do not necessarily represent those of their affiliated organizations, or those of the publisher, the editors and the reviewers. Any product that may be evaluated in this article, or claim that may be made by its manufacturer, is not guaranteed or endorsed by the publisher.
